# Anti-Inflammatory and Anti-Oxidative Effects of Polysaccharides Extracted from Unripe *Carica papaya* L. Fruit

**DOI:** 10.3390/antiox12081506

**Published:** 2023-07-27

**Authors:** Ting-Yun Lin, Yun-Ting Wu, Hui-Ju Chang, Chun-Chen Huang, Kuan-Chen Cheng, Hsien-Yi Hsu, Chang-Wei Hsieh

**Affiliations:** 1 Department of Food Science and Biotechnology, National Chung Hsing University, Taichung City 402202, Taiwan; g109043105@mail.nchu.edu.tw (T.-Y.L.); g110043404@mail.nchu.edu.tw (Y.-T.W.); z23590819@dragon.nchu.edu.tw (C.-C.H.); 2 Department of Taiwan Seed Improvement and Propagation Station, Council of Agriculture, Executive Yuan, Taichung City 426017, Taiwan; tracyc@tss.gov.tw; 3 Institute of Biotechnology, National Taiwan University, Taipei 10617, Taiwan; kccheng@ntu.edu.tw; 4 Graduate Institute of Food Science Technology, National Taiwan University, Taipei 10617, Taiwan; 5 Department of Optometry, Asia University, Taichung City 413305, Taiwan; 6 Department of Medical Research, China Medical University Hospital, Taichung City 404333, Taiwan; 7 Shenzhen Research Institute, City University of Hong Kong, Shenzhen 518057, China; sam.hyhsu@cityu.edu.hk; 8 Department of Materials Science and Engineering, City University of Hong Kong, Hong Kong 999077, China

**Keywords:** *Carica papaya* L., antioxidative, anti-inflammatory

## Abstract

This study evaluated the antioxidative and anti-inflammatory activities of polysaccharides extracted from unripe *Carica papaya* L. (papaya) fruit. Three papaya polysaccharide (PP) fractions, namely PP-1, PP-2, and PP-3, with molecular weights of 2252, 2448, and 3741 kDa, containing abundant xylose, galacturonic acid, and mannose constituents, respectively, were obtained using diethylaminoethyl–Sepharose™ anion exchange chromatography. The antioxidant capacity of the PPs, hydroxyl radical scavenging assay, ferrous ion-chelating assay, and reducing power assay revealed that the PP-3 fraction had the highest antioxidant activity, with an EC_50_ (the concentration for 50% of the maximal effect) of 0.96 mg/mL, EC_50_ of 0.10 mg/mL, and Abs700 nm of 1.581 for the hydroxyl radical scavenging assay, ferrous ion-chelating assay, and reducing power assay, respectively. In addition, PP-3 significantly decreased reactive oxygen species production by 45.3%, NF-κB activation by 32.0%, and tumor necrosis factor-alpha and interleukin-6 generation by 33.5% and 34.4%, respectively, in H_2_O_2_-induced human epidermal keratinocytes. PP-3 exerts potent antioxidative and anti-inflammatory effects; thus, it is a potential biofunctional ingredient in the cosmetic industry.

## 1. Introduction

Plant polysaccharides are biological polymers with immunomodulatory [1,2], anti-inflammatory [3,4], and antioxidant [5,6] properties. A previous study showed that polysaccharides extracted from *Morus nigra* L. protected PC-12 cells from H_2_O_2_-induced oxidative damage [7]. *Siraitia grosvenorii* polysaccharides are key regulators of NF-κB activation and can reduce the protein expression of tumor necrosis factor-alpha (TNF-α) and interleukin (IL)-6 and consequently delay inflammation [8]. Barley (*Hordeum vulgare* L.) polysaccharides have been reported to inhibit the translocation of NF-κB from the cytoplasm to the nucleus [9]. Plant species display distinct polysaccharide functions; therefore, clarifying their biofunctions in common fruits may increase the added value of crops.

Papaya is the fourth most globally traded tropical fruit for its nutritional value [10]. Papaya fruits, peels, seeds, roots, and leaves exhibit nutritional and therapeutic significance; thus, papaya has been extensively used in folk medicine [11,12]. Unripe papaya extracts have been shown to exhibit dose-dependent reactive oxygen species (ROS) scavenging activities, which can reduce NF-κB activation and enhance antioxidant defense systems by increasing superoxide dismutase and catalase activities [13]. In addition, the increased antioxidant enzyme activity in papaya extracts can improve wound healing rates and inhibit wound inflammation by regulating the expression of proinflammatory factors [14]. Papaya extracts have also been demonstrated to inhibit NF-κB activation and dysregulate Nrf2, which is crucial for maintaining redox homeostasis, thereby delaying skin aging [15]. Furthermore, papaya polysaccharides (PPs) have been shown to possess skin moisture-preserving activities [16]. Differentially charged polysaccharide fractions can be efficiently separated and accurately purified using anion exchange column chromatography [17].

The human skin provides protection from the external environment [18]. Keratinocytes are predominant skin cells that secrete cytokines with proinflammatory activities upon stimulation, leading to skin diseases such as psoriasis, contact dermatitis, and atopic dermatitis [19]. Consequently, such infections can disrupt the skin epithelial structure [20]. ROS are associated with inflammatory response through several mechanisms, such as modulating related signaling pathways and altering cellular metabolism [13]. Dexamethasone (DEX) is a widely used glucocorticoid for controlling skin inflammation; however, such glucocorticoids are associated with several side effects [21], which necessitate the development of novel anti-inflammatory skin disease agents. Although papaya is known to be effective in treating numerous skin and inflammatory conditions, only a few reports have explored the chemical and physical properties of unripe PPs for application in the cosmetic industry. Thus, this study investigated the chemical composition, antioxidant activity, and anti-inflammatory effect of unripe PPs on keratinocytes to identify novel cosmetic ingredients.

## 2. Materials and Methods

### 2.1. Chemicals and Reagents

*Carica papaya* L. cv. Risheng was cultivated in Tainan County, Taiwan, and purchased from Fushoushan Farm (Taichung, Taiwan). Ion-exchange resin diethylaminoethyl (DEAE)–Sepharose™ CL-6B was purchased from GE Healthcare Life Sciences (Uppsala, Sweden). The fluorescein-labeled dye H_2_DCFDA and thiazolyl blue tetrazolium bromide were purchased from Sigma-Aldrich (St. Louis, MO, USA). Hydrogen peroxide was purchased from Echo Chemical Co., LTD (Taiwan). The NFκB-p65 polyclonal antibody (E-AB-32233), GAPDH polyclonal antibody (E-AB-40337), and goat anti-rabbit IgG (H + L; peroxidase/HRP conjugated; E-AB-1003) were purchased from Elabscience (Houston, TX, USA).

### 2.2. Extraction of Polysaccharides

Crude polysaccharides from unripe *C. papaya* L. cv. Risheng (PP-URS) were extracted using a previously reported method [16] with slight modifications. The dry papaya fruit was first immersed in 80% (*v*/*v*) ethanol at a solid-to-liquid ratio of 1:10 (*w*/*v*) at 80 °C for 4 h to remove fat-soluble substances, and the precipitates were dispersed in distilled water at a solid-to-liquid ratio of 1:25 (*w*/*v*) at 75 °C for 40 min. The extraction solutions were concentrated using a rotary evaporator and were precipitated with ethanol at 4 °C for 24 h to obtain a final concentration of 80% (*v*/*v*). The precipitates were collected, centrifuged at 9000× *g* for 15 min, lyophilized, and tagged as PP-URS.

### 2.3. Isolation and Purification of PPs

The unripe papaya crude polysaccharide solution (PP-URS) was dialyzed for 48 h through a dialysis membrane (cellulose tube, 30/32; UC30-32-100; molecular weight cutoff of 12–14 kDa; Sanko Pure Chemical Industries, Ichinomiya, Japan). The macromolecular PP-URS was loaded onto a DEAE–Sepharose™ CL-6B gel column (3.8 cm × 9.0 cm) after lyophilization for 72 h. The column was eluted with a sodium chloride solution (0.1, 0.3, and 0.5 M) at a flow rate of 1.0 mL/min. The 0–70 tubes were eluted with distilled water (PP-1), 71–140 tubes were eluted with 0.1 M of a NaCl solution (PP-2), 140–210 tubes were eluted with 0.3 M of a NaCl solution (PP-3), and 211–280 tubes were eluted with 0.5 M of a NaCl solution. Overall, 10 mL of eluent fractions were collected automatically, and the polysaccharide content of each tube was analyzed using the phenol–sulfuric acid method [16]. The fraction eluted with 0.5 M of NaCl could not be obtained because of low absorbance at 490 nm. The eluents were dialyzed and lyophilized to obtain three polysaccharide fractions, namely PP-1, PP-2, and PP-3.

### 2.4. Chemical Analysis

The polysaccharide, uronic acid, protein, and polyphenol contents of the three polysaccharide fractions were estimated using the phenol–sulfuric acid method with d-glucose (99.5%, Sigma-Aldrich, St. Louis, MO, USA) as the standard [16], the *meta*-hydroxydiphenyl method with galacturonic acid (97.0%, Sigma-Aldrich) as the standard [22], the Bradford method with bovine serum albumin (98.0%, Apolo, New York, NY, USA) as the standard [16], and the Folin–Ciocalteu method with gallic acid as the standard, respectively [23].

### 2.5. Determination of Monosaccharide Components

High-performance liquid chromatography (HPLC) was used to detect monosaccharide components after acid hydrolysis and derivatization, as described previously [24]. Approximately 50 mg of the polysaccharide fractions was hydrolyzed in 10 mL of trifluoroacetic acid (TFA) at 110 °C for 2 h. Subsequently, the dried hydrolysates were mixed with 20.8 mL of 0.3 M of sodium hydroxide. Then, 10 mL of a 0.5 M PMP methanol solution was added at 70 °C for 30 min, followed by the addition of 0.3 M of hydrochloric acid. The mixture was evaporated to dryness and then mixed with chloroform. The chloroform phase was discarded after shaking, and the PMP derivative solution was filtered through a 0.45-μm organic syringe filter for HPLC analysis. The HPLC system (Hitachi, Chiyoda City, Japan) consisted of a quaternary pump (Chromaster 5110), auto sampler (Chromaster 5260), and UV–Vis detector (Chromaster 5420) at a wavelength of 245 nm. The column (Mightysil RP-18 GP column, 5 μm, 4.6 mm × 250 mm) was eluted with 83% of a phosphate buffer (pH 6.7, 0.1 M) and 17% acetonitrile (1.0 mL/min).

### 2.6. Determination of the Molecular Weight of Polysaccharide Fractions

Molecular weight distribution was determined using HPLC (Hitachi) via a Shodex OH pak SB-806M HQ (8 mm × 300 mm, Shodex, Showa Denko America, New York, NY, USA) matching the refractive index detector (Hitachi chromaster 5450), as described previously [25]. The column was eluted with the mobile phase of water at a flow rate of 1.0 mL/min. The polysaccharide fractions were dissolved in deionized water, and dextran standards with different molecular weights were used as standards.

### 2.7. Fourier Transform Infrared (FTIR) Spectroscopic Analysis

Spectroscopic analysis of 2 mg of polysaccharide fractions was performed using an FTIR spectrometer (Nicolet iS5, Thermo, Waltham, MA, USA), as described previously [16], in the range of 4000–600 cm^−1^.

### 2.8. Determination of Antioxidant Capacities

#### 2.8.1. Hydroxyl Radical Scavenging Activity

Overall, 50 μL of polysaccharide fractions (0–5 mg/mL) was mixed with 50 μL of 9 mM of sodium salicylate, 50 μL of 9 mM of iron (II) sulfate heptahydrate, and 50 μL of hydrogen peroxide (0.025%, *v*/*v*) at 37 °C for 30 min [26]. Then, the hydroxyl radical scavenging activity was measured at 510 nm, with Vit C as the positive control. The hydroxyl radical scavenging rate of antioxidants was calculated as follows:

Hydroxyl radical scavenging rate (%) = [(Ac − As)/Ac] × 100% (1)

where Ac is the absorbance of the control mixture, and As is the absorbance of the sample mixture.

#### 2.8.2. Chelating Capacity of Ferrous Ions

Overall, 250 μL of polysaccharide fractions (0–1 mg/mL) was mixed with 10 μL of 2 mM of iron (III) chloride and 20 μL of 5 mM of ferrozine for 10 min [27]. Then, the chelating capacity of ferrous ions was measured at 562 nm, with EDTA-2Na as the positive control. The chelating rate of ferrous ions was calculated as follows:

Ferrous ions chelating rate (%) = [(Ac − As)/Ac] × 100%(2)

where Ac is the absorbance of the control mixture, and As is the absorbance of the sample mixture.

#### 2.8.3. Reducing Power Assay

Approximately 400 μL of the polysaccharide fractions (0–5 mg/mL) was mixed with 100 μL of potassium ferricyanide (III, 1%, *w*/*v*) and 100 μL of 0.2 M of a phosphate buffer, and the mixture was incubated at 50 °C for 30 min. Subsequently, 100 µL of trichloroacetic acid (10%, *v*/*v*) was added to the mixture to terminate the reaction, and the mixture was subjected to centrifugation at 6000 rpm for 10 min. A 0.25 mL aliquot of the supernatant was mixed with 0.05 mL of 0.1% ferric chloride [28], and the absorbance of the mixture was measured at 700 nm, with Vit C as the positive control.

### 2.9. Cell Viability Assay

A cell viability assay was performed as previously described [29]. H_2_O_2_-induced human epidermal keratinocytes (HaCaTs) were seeded into 96-well plates (2 × 10^4^ cThls/mL) for 24 h. A medium (200 μL) containing various concentrations of PPs (50, 100, 200, 400, 600, and 800 μg/mL) or H_2_O_2_ (50, 100, 200, 300, 400, 500, 600, 700, 800, 900, and 1,000 μM) was added to the wells. After 24 h of incubation, the medium was mixed with 200 μL of an MTT solution (5 mg/mL, *w*/*v*) and incubated for 2 h. Then, DMSO (200 μL) was added to the mixture, and the absorbance was measured at 570 nm. The cell survival rate was calculated as follows:

Cell survival rate (%) = A_treated group_ ⁄rA_blank_ × 100% (3)

### 2.10. Intracellular ROS Production Assay

The content of intracellular ROS was determined as previously described with few modifications [30]. HaCaTs were seeded into 6-well plates (3 × 10^5^ cells/mL) for 24 h and pretreated with PPs for 24 h, followed by treatment with 2 mL of 600 μM of H_2_O_2_ for 4 h. Next, DCFH-DA (5 μM) was added under dark conditions at 37 °C for 30 min, and intracellular ROS production was measured using a 20× fluorescence microscope (Olympus IX51, Tokyo, Japan). The mean fluorescence intensity was analyzed using Image J software v.1.54d (National Institutes of Health, Bethesda, MD, USA).

### 2.11. Western Blot Analysis

HaCaTs were lysed using a 1× RIPA buffer in an ice-water bath to obtain protein. The protein samples were separated via electrophoresis on an 8% SDS–PAGE gel and then transferred to nitrocellulose filter membranes. Subsequently, the membranes were blocked with 5% nonfat milk in a TBST buffer at room temperature for 1 h. Finally, the membranes were incubated with primary antibodies against NFκB-p65 (1:500), phospho-NFκB-p65 (1:500), and GADPH (1:500) at 4 °C for 24 h, followed by incubation with secondary antibodies (1:1000) at room temperature for 1 h. An ECL immunosensor (Amersham Biosciences, Buckinghamshire, UK) was used for the detection of chemiluminescence.

### 2.12. Evaluation of IL-6 and TNF-α Levels in HaCaTs

HaCaTs at a concentration of 2 × 10^6^ cells/well were added to a 10-cm^2^ dish and were induced using 600 μM H_2_O_2_. PP-3-treated and untreated cells were incubated at 37 °C for 24 h. The levels of IL-6 and TNF-α were quantified using an ELISA quantitative kit (Bender Medsystems, Burlingame, CA, USA) in accordance with the manufacturer’s protocol.

### 2.13. Statistical Analysis

Comparison between treatment groups was performed using SPSS 20, and all data were represented as the mean ± standard deviation (SD, *n* = 3). Statistical significance at *p*-values of <0.05 was assessed using one-way analysis of variance and Duncan’s multiple range tests.

## 3. Results

### 3.1. Purification of Crude PP-URS

**Figure 1:**
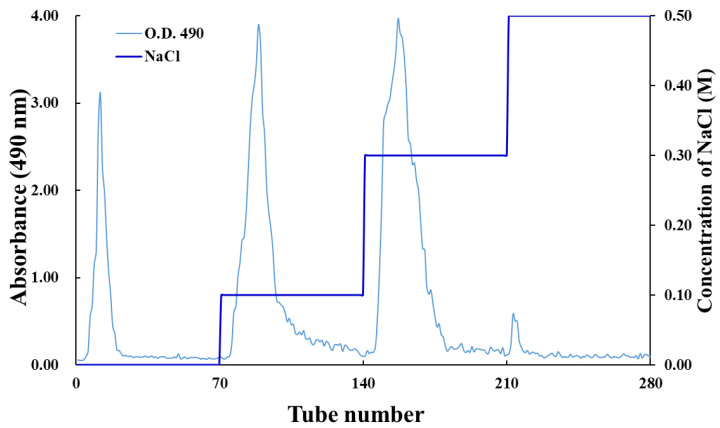
Fractionation and purification profiles of PP-URS on diethylaminoethyl–Sepharose™ CL-6B column.

As shown in Figure 1, the ion-exchange chromatographic analysis of crude PP-URS polysaccharides revealed three mean peaks, namely PP-1, PP-2, and PP-3 [31]. The contents of the polysaccharides, uronic acid, protein, and polyphenols in the purified fractions are shown in Table 1. The recovery rates of the purified polysaccharides were 2.76%, 12.47%, and 31.21% for PP-1, PP-2, and PP-3, respectively, with a narrow variation of 81.60–84.90% in the content of isolated fractions. The uronic acid content in the PP-2 and PP-3 fractions was significantly higher than that in the PP-1 fraction, which is consistent with a previous study [32]. Similarly, based on anionic exchange resin, *Cyclocarya paliurus* (Batal) Iljinskaja polysaccharides had a high total content of carbohydrates (79.6%), followed by uronic acids (20.2%), and a relatively low content of protein (8.44%) [33,34].

### 3.2. Composition and Molecular Weights of Polysaccharide Fractions

The constituents of monosaccharides are associated with their biological activities [35]. As shown in Table 2, the three identified PPs were heteropolysaccharides. Seven monosaccharides were identified from PP-1, among which galacturonic acid and xylose exhibited a high molar ratio. Six monosaccharides were identified from PP-2 and PP-3. PP-2 primarily contained galacturonic acid, and PP-3 had a high molar ratio of mannose and galacturonic acid. PP-2 and PP-3 contained more acidic polysaccharides, consistent with the uronic acid contents shown in Table 1. The contents of galacturonic acid, rhamnose, and arabinose in plant polysaccharides are closely linked to their antioxidant activity [35,36,37]. Polysaccharides with high mannose contents have been shown to possess remarkable free radical scavenging activity, with strong activity at a mannose-to-galactose ratio of approximately 1.8:1; however, the detailed mechanism underlying this effect requires further investigation [38].

As shown in Table 2, the molecular weight of the three polysaccharide fractions ranged from 2252 to 3741 kDa, which was slightly different from the previously reported range of 2510–2650 kDa obtained from papaya cv. Waimanalo used in anion exchange column elution [16], possibly due to the different papaya varieties used in that study. These results demonstrated that PP-3 had a molecular weight of 3741 kDa and was rich in galacturonic acid and mannose. The potential application of unripe PPs in the manufacturing of cosmetics has been previously speculated [35,39].

### 3.3. Determination of the Functional Groups of PPs

**Figure 2:**
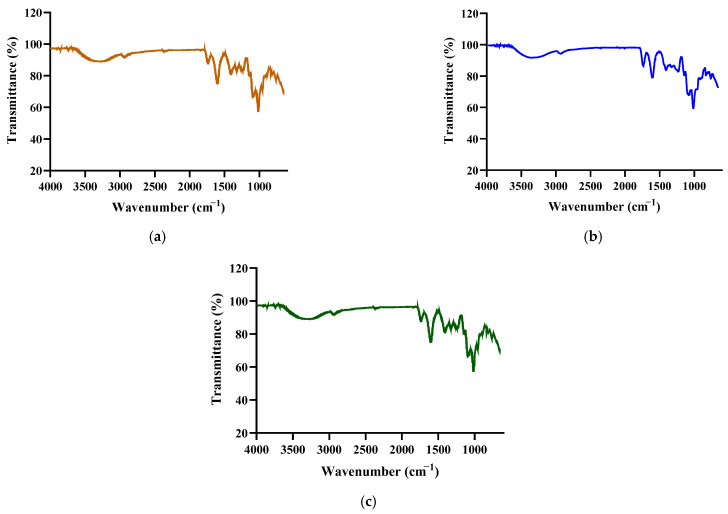
Characterization of the functional groups of (**a**) PP-1, (**b**) PP-2, and (**c**) PP-3.

As shown in Figure 2, the three polysaccharide fractions exhibited typical peaks [40]. The peak at 3343 cm^−1^ was attributed to O–H stretching vibration [41,42]. The absorption peak at 2973 cm^−1^ indicated that polysaccharides had C–H vibration. The peak around 1735 cm^−1^ represented C=O asymmetric stretching vibration. The absorption peak at 1602 cm^−1^ was assigned to the C=O asymmetric stretching of −COO [28,43]. Moreover, the peak at 1415 cm^−1^ was attributed to C–O stretching and O–H deformation vibrations [40,44]. The strong peak at 1073 cm^−1^ indicated C–O–C and C–O–H stretching vibrations [45]. The FTIR spectra indicated that the three PPs had typical absorption patterns, and differences in their patterns did not influence the functional group.

### 3.4. Analysis of the Antioxidant Activity

#### 3.4.1. Hydroxyl Radical Scavenging Activity of PPs

**Figure 3:**
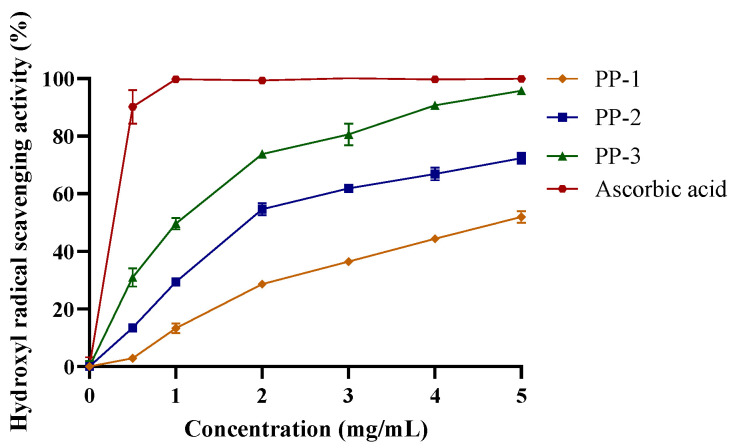
Hydroxyl radical scavenging activity of PPs. Data are represented as mean ± SD of three independent experiments.

^•^OH can react with biomolecules and cause tissue damage or cell death [46]. Figure 3 shows that the hydroxyl radical scavenging activity of PPs increased in a concentration-dependent manner, with PP-3 exhibiting the strongest scavenging activity. Cells can scavenge hydroxyl radicals through various mechanisms, such as prevention of hydrogen abstraction, decomposition of peroxides, and suppression of hydroxyl radical synthesis [47]. PP-3 could inhibit ^•^OH generation by chelating ferrous ions [23].

#### 3.4.2. Ferrous Ion-Chelating Capacity of PPs

**Figure 4:**
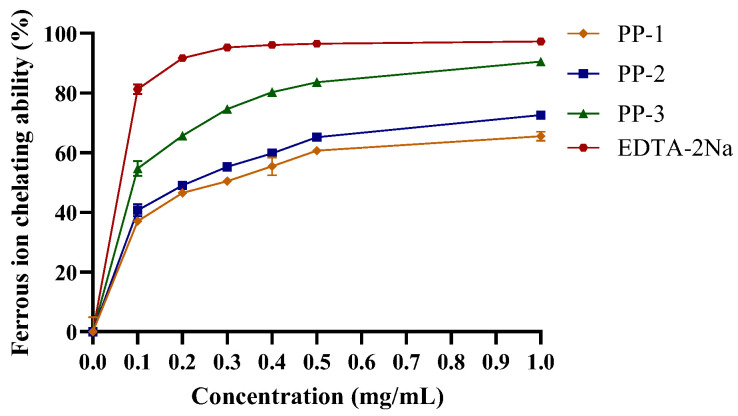
Ferrous ion-chelating capacity of PPs. Data are represented as the mean ± SD of three independent experiments.

The electron transformation of ferrous ions (Fe^2+^) is associated with numerous physiological processes, such as enzymatic reactions, redox reactions, and cellular metabolism [48]. As shown in Figure 4, the ferrous ion-chelating capacity of PPs was concentration-dependent, with an EC_50_ (the concentration for 50% of the maximal effect) of PP-1, PP-2, and PP-3 being 0.10, 0.21, and 0.20 mg/mL, respectively. Notably, the patterns of hydroxyl radical scavenging activity were consistent with the ferrous ion-chelating capacity of the PPs, which is in agreement with previous findings [49]. The high molecular weight of PP-3 indicated that it could generate more hydroxyl groups that could react with radicals to terminate their chain reaction [35,50].

#### 3.4.3. PPs Exhibit Reducing Power in a Concentration-Dependent Manner

**Figure 5:**
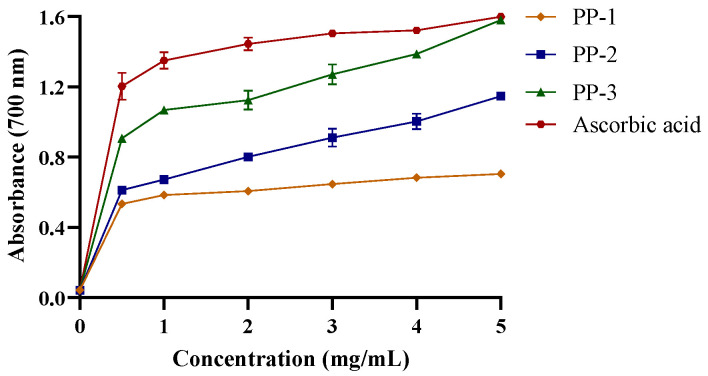
Reducing power of PPs. Data are represented as the mean ± SD of three independent experiments.

Reductones are closely associated with the reducing the properties of polysaccharides [51]. As shown in Figure 5, PP-1, PP-2, and PP-3 exhibited reducing power in a concentration-dependent manner, with absorbances of 0.705, 1.147, and 1.581, respectively, at 5.0 mg/mL. Previous studies have determined the reducing power based on electrophilic functional groups [52,53,54]. In this study, PP-3, with high uronic acid content and molecular weight, showed notable antioxidant activity.

### 3.5. PP-3 Improves Cell Viability by Reversing the Antiproliferative Effect of ROS

**Figure 6:**
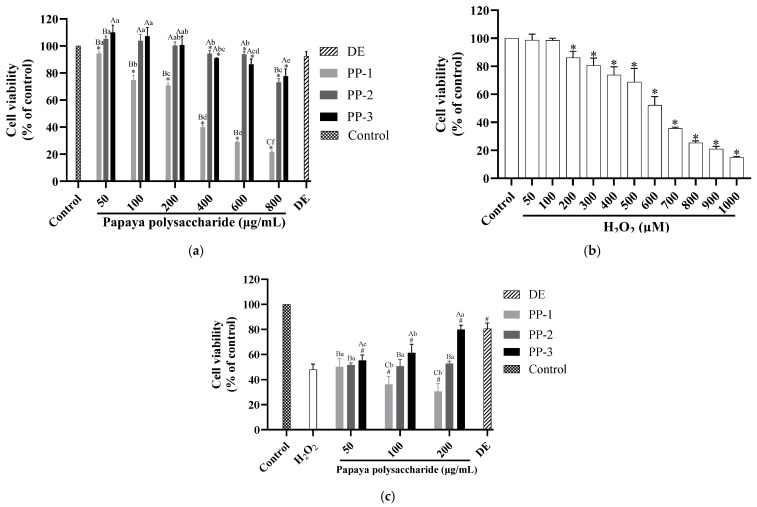
(**a**) Cytotoxicity of HaCaTs incubated with PPs. (**b**) Cytotoxicity of HaCaTs incubated with H_2_O_2_. * indicates statistical significance at *p* < 0.05 relative to the control group. (**c**) Cytotoxicity of HaCaTs incubated with PPs at 50, 100, and 200 μg/mL for 24 h, followed by treatment with 600 μM H_2_O_2_. The results are represented as the means ± SD of triplicate independent experiments (*n* = 3). Different uppercase letters represent significant differences of samples at the same concentration at *p* < 0.05. Different lowercase letters represent significant differences in the same sample at different concentrations at *p* < 0.05. # indicates significant differences relative to the H_2_O_2_ group at *p* < 0.05.

The cytotoxicity of PPs on HaCaTs was assessed using an MTT assay [55]. Figure 6a shows that PP-2 and PP-3 showed no toxicity to HaCaTs at concentrations <200 μg/mL. Consequently, three doses of PPs (50, 100, and 200 μg/mL) were used to analyze their alleviation capacity against H_2_O_2_-induced oxidative damage. HaCaTs were pretreated with different concentrations of H_2_O_2_ (0–1000 μM) to obtain a suitable cell damage model. Figure 6b shows that the viability of the HaCaTs decreased with the H_2_O_2_ treatment. In addition, the viability of the HaCaTs incubated with 200 μM of H_2_O_2_ decreased significantly, and cells incubated with 600 μM of H_2_O_2_ had a viability rate of only 52.23%. Thus, this concentration was selected for inducing cell oxidative damage. As shown in Figure 6c, the effect of PPs on HaCaT viability under oxidative damage decreased significantly to 48% compared with the control. In contrast, different concentrations of PP-3 could reverse the antiproliferative effect of H_2_O_2_ on HaCaTs (*p* < 0.05), with its effects at 200 μg/mL showing no significant difference compared with that of the positive control (DEX) at 20 μg/mL. Therefore, PP-3 at the abovementioned three concentrations was used for further experiments.

### 3.6. PP-3 Mitigates Intracellular ROS Production

**Figure 7:**
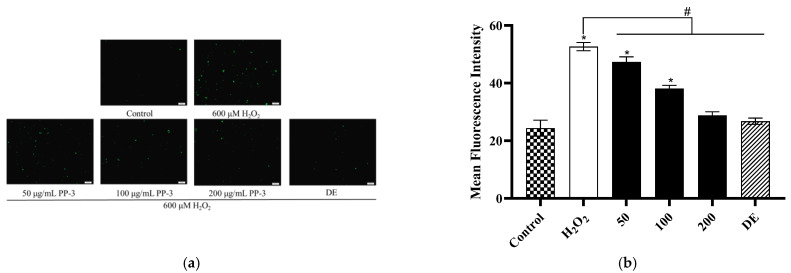
(**a**) Effects of PP-3 on H_2_O_2_-induced ROS production in HaCaTs. Scale bar: 100 μm. (**b**) Quantitative analysis of ROS content in the control or PP-3 and H_2_O_2_-treated HaCaTs. ROS content was detected by DCFH-DA and examined with 20× fluorescence microscope. The intracellular green DCF fluorescence indicates as a result of ROS production. The results are represented as the mean ± SD of three independent experiments (*n* = 3). * indicates significant differences relative to the control group at *p* < 0.05. # indicates significant differences relative to the H_2_O_2_ group at *p* < 0.05.

ROS generation is widely used to predict the level of cell oxidative stress [29]. After treatment with 600 μM of H_2_O_2_, intracellular ROS production was determined in H_2_O_2_-induced cells to understand the effects of PPs on oxidative damage. As shown in Figure 7a, the oxidative damage of the H_2_O_2_-induced group was stronger than that of the control group, whereas pretreatment with PP-3 and DEX showed an evident decrease in fluorescent intensity. Figure 7b shows that the inhibition rate of PP-3 at 50, 100, and 200 μg/mL was 10.1%, 27.6%, and 45.3%, respectively. The effects of PP-3 at 200 μg/mL were not significantly different (*p* > 0.05) from that of the control and DEX groups at 20 μg/mL, indicating that PP-3 effectively reduces ROS generation. This finding demonstrates that PP-3 is a candidate antioxidative agent that can be used for the development of skincare products.

### 3.7. PP-3 Mediates the Reduction of NF-κB Phosphorylation

**Figure 8:**
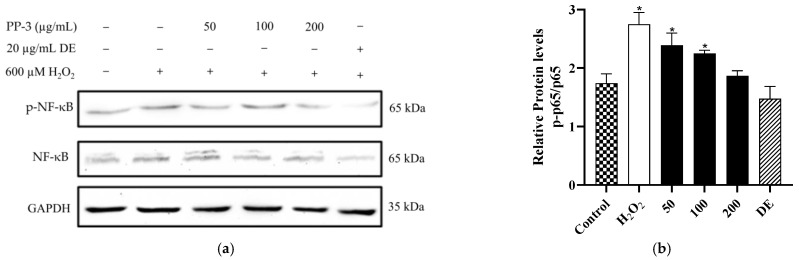
(**a**) Effects of PP-3 on the expression of NF-κb and p-NF-κb in H_2_O_2_-induced HaCaTs. (**b**) Quantitative analysis of the effect of PP-3 on the expression of the p-NF-κB/NF-κB ratio in H_2_O_2_-induced HaCaTs. Relative changes in protein intensities in the Western blot were quantified using densitometric analysis, with GAPDH as an internal control, and were then presented as bar plots. The results are represented as the mean ± SD of three independent experiments (*n* = 3). * indicates significant difference relative to the control group at *p* < 0.05.

NF-κB is the major transcription factor that regulates numerous cell biochemical reactions [56]. After external stimulation, NF-κB is activated, followed by the phosphorylation of serine residues in specific positions of IκB and transcriptional regulation of target genes, leading to the production of proinflammatory cytokines, such as TNF-α and IL-6 [57,58]. The expression level of p-p65/p65 in the H_2_O_2_-induced group increased significantly (*p* < 0.05) (Figure 8a). In contrast, compared with the control, pretreatment with 50, 100, and 200 μg/mL of PP-3 significantly decreased the expression level of p-p65/p65, reducing the phosphorylation ratio to 13.1%, 18.1%, and 32.0%, respectively, as shown in Figure 8b. These results indicated that PP-3 protects HaCaTs by regulating the expression level of NF-κB, a key regulator of inflammatory response.

### 3.8. PP-3 Regulates the Level of Inflammatory Cytokines

**Figure 9:**
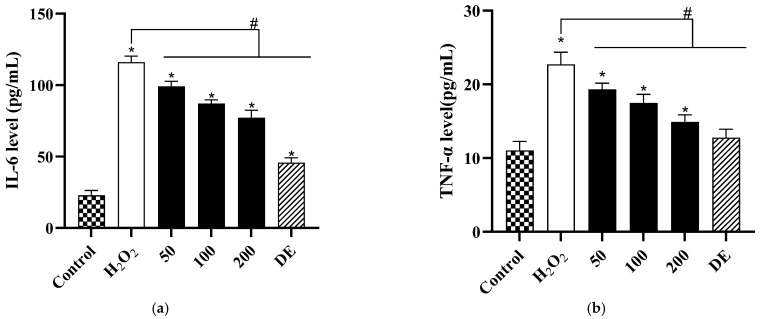
Effects of PP-3 on (**a**) IL-6 and (**b**) TNF-α production in H_2_O_2_-induced HaCaTs. The results are represented as mean ± SD of three independent experiments (*n* = 3). * indicates significant differences relative to the control group at *p* < 0.05. # indicates significant differences relative to the H_2_O_2_ group at *p* < 0.05.

As shown in Figure 9a, compared with the control group, the level of IL-6 in the H_2_O_2_-induced group increased sharply from 22.92 to 116.06 pg/mL. In contrast, pretreatment with PP-3 at 50, 100, and 200 μg/mL markedly decreased the IL-6 levels to 99.24, 87.12, and 77.19 pg/mL, respectively. In addition, TNF-α production increased from 11.04 to 22.71 pg/mL in the H_2_O_2_-induced group (Figure 9b), whereas TNF-α levels markedly decreased to 19.31, 17.50, and 14.89 pg/mL after pretreatment with 50, 100, and 200 μg/mL PP-3, respectively. Intercellular ROS production, NF-κB phosphorylation, and inflammatory cytokine levels consistently showed that the negative effects of induced H_2_O_2_ on HaCaTs were alleviated by PP-3. Moreover, PP-3 at 200 μg/mL significantly inhibited ROS generation and accumulation, as well as NF-κB phosphorylation, followed by the reduction of IL-6 and TNF-α production. Collectively, these results demonstrated that unripe PPs exert anti-inflammatory effects; thus, they can be used as a key raw material in the cosmetic industry.

## 4. Discussion

Excessive ROS is the fundamental cause of several chronic diseases. ROS can induce oxidative stress and cause cell damage by attacking cell membranes and regulating the expression of relevant proteins related to oxidative damage. Long-term exposure of the human skin to environmental stimuli, such as ultraviolet light and air pollution, and the natural aging of the skin can lead to ROS production and accumulation [59]. Excessive ROS can enhance vasodilation and promote the migration of inflammatory cells, leading to tissue damage in the skin [60]. In addition, ROS can increase tyrosinase expression and cause collagen degradation, thereby resulting in changes in the appearance, such as inflammation, dullness, dehydration, pigmentation, and loss of functionality [61]. In a previous study, *Typha angustifolia* polysaccharides obtained through anionic exchange resin showed an evident decrease in fluorescent intensity by 26% at 200 μg/mL, with a PP-3 inhibition rate of 45.3% [4]. PP-3 can effectively inhibit intracellular ROS owing to its antioxidant capacity. Polysaccharides with high uronic acid content are likely to provide hydrogen atoms, exerting optimal antioxidant effect [62]. In this study, the uronic acid content followed the order of PP-3 > PP-2 > PP-1. Therefore, the decrease in fluorescent intensity may be due to the high uronic acid content. Polysaccharides with a high molecular weight possess more active functional groups, thereby exhibiting better biological activity [39]. Furthermore, the antioxidant analysis revealed that PP-3 possessed notable antioxidant activity. The results of the present study indicated that PP3 obtained through anionic exchange resin can ameliorate H_2_O_2_-induced oxidative stress and can be used for the development of skincare products.

In our previous research, we compared the structural characteristics and ABTS^+^ scavenging activity of ripe and unripe PPs. Based on functional group analysis, PPs extracted at different maturity levels showed similar chemical structures. They all possessed the typical structures of polysaccharides, such as C–H, O–H, and C=O groups. PPs extracted at different maturity levels did not influence the functional groups. With regard to antioxidant activity, the ABTS^+^ scavenging assay revealed that unripe PPs (EC_50_ = 6.26 mg/mL) exhibited higher antioxidant activity than ripe PPs (EC_50_ = 9.21 mg/mL).

PP-1, PP-2, and PP-3 were fractioned using different concentrations of the NaCl solution and the ion-exchange resin diethylaminoethyl (DEAE–Sepharose™ CL-6B) column. The fractionation and purification profiles are shown in Figure 1. The contents of the polysaccharides, proteins, and uronic acid are shown in Table 1. The constituent monosaccharides and molecular weights of the PPs are shown in Table 2. In the present study, PP-3 showed the best antioxidant and anti-inflammatory activities compared with PP-1 and PP-2 because PP-3 is rich in uronic acid (76.69%) and has a high molecular weight (3741 kDa).

The antioxidant capacity of the PPs, hydroxyl radical scavenging assay, ferrous ion-chelating assay, and reducing power assay revealed that the PP-3 fraction had the highest antioxidant activity, with an EC_50_ of 0.96 mg/mL, an EC_50_ of 0.10 mg/mL, and an Abs700 nm of 1.581 for the hydroxyl radical scavenging assay, ferrous ion-chelating assay, and reducing power assay, respectively.

Many skin diseases are associated with an imbalanced immune response [63]. Previous studies have indicated an increased expression of NF-κB in inflammatory skin diseases, leading to symptoms such as skin cracking, roughness, redness, and scaling, which affect the quality of life of patients [32,64]. NF-κB is considered a key transcriptional regulator of inflammation-related genes, and the phosphorylation level of NF-κB plays a crucial role in inflammation regulation [65]. When NF-κB is activated and translocated to the nucleus, it binds to specific DNA sites, resulting in the production of proinflammatory cytokines [57]. An appropriate level of proinflammatory cytokines is essential to maintain the internal stability of keratinocytes. However, during the diseased state of the keratinocytes, the signaling pathways shift from maintenance to regulation, thereby increasing the level of proinflammatory cytokines, such as TNF-α and IL-6 [13]. In this study, pretreatment with PP-3 significantly decreased the expression level of p-p65/p65, reduced the phosphorylation ratio to 32.0%, and decreased IL-6 and TNF-α production to 33.5% and 34.4% at 200 μg/mL relative to the control. PP3 has been shown to effectively inhibit ROS generation and accumulation, suppressing NF-κB phosphorylation to reduce IL-6 and TNF-α production. We hypothesized that *C. papaya* polysaccharides could protect HaCaTs under oxidative stress owing to their favorable antioxidant and anti-inflammatory abilities to scavenge intracellular ROS.

## 5. Conclusions

We conducted a comprehensive characterization study based on a chemical composition analysis and the anti-inflammatory activity of polysaccharides extracted from papaya. The analysis of polysaccharide fractions revealed three mean peaks, namely PP-1, PP-2, and PP-3, with varied molar ratios and molecular weights. Of the three fractions, PP-3 demonstrated the highest antioxidant activity. The anti-inflammatory assay indicated that PP-3 pretreatment significantly suppressed ROS accumulation, NF-κB activation, and proinflammatory cytokine generation in H_2_O_2_-induced HaCaTs. Thus, the anti-inflammatory effects of PP-3 should be further investigated to validate its potential application as a cosmetic ingredient.

## Figures and Tables

**Table 1:** Fractionation and purification profiles of PPs.

		Chemical Composition (%)			
	Yield	Polysaccharide	Protein	Uronic Acid	Polyphenol
PP-1	2.76%	84.90 ± 1.70 ^a^	N.D.	43.49 ± 0.06 ^b^	0.16 ± 0.01 ^b^
PP-2	12.47%	81.60 ± 0.73 ^b^	N.D.	76.52 ± 1.39 ^a^	0.35 ± 0.01 ^a^
PP-3	31.21%	83.51 ± 0.64 ^ab^	N.D.	76.69 ± 0.57 ^a^	0.06 ± 0.01 ^c^

Data are represented as mean ± SD of three independent replicates (*n* = 3). Lowercase letters indicate statistical significance at *p*-values of <0.05 in the same column. N.D., not detected.

**Table 2:** Constituent monosaccharides and molecular weights of PPs.

Constituent Monosaccharides (Molar Ratios)								Molecular Weight (kDa)
	Mannose	Rhamnose	Galacturonic Acid	Glucose	Galactose	Xylose	Arabinose	
PP-1	1.58	1.00	2.62	1.12	1.81	2.79	0.99	2252
PP-2	N.D.	1.00	3.09	2.03	2.22	2.21	1.05	2448
PP-3	4.47	1.00	2.99	2.06	2.47	N.D.	2.05	3741

N.D., not detected.

## Data Availability

Data is contained within the article.
